# Editorial: Methods in neuro-oncology and neurosurgical oncology

**DOI:** 10.3389/fonc.2023.1213965

**Published:** 2023-06-05

**Authors:** Matteo Zoli, Gueliz Acker

**Affiliations:** ^1^ Department of Biomedical and Neuromotor Sciences (DIBINEM), University of Bologna, Bologna, Italy; ^2^ IRCCS Istituto delle Scienze Neurologiche di Bologna, Programma Neurochirurgia Ipofisi – Pituitary Unit, Bologna, Italy; ^3^ Department of Neurosurgery, Charité-Universitätsmedizin Berlin (Corporate Member of Freie Universität Berlin, Humboldt-Universität zu Berlin, and Berlin Institute of Health), Berlin, Germany; ^4^ Department of Radiation Oncology and Radiotherapy, Charité-Universitätsmedizin Berlin (Corporate Member of Freie Universität Berlin, Humboldt-Universität zu Berlin, and Berlin Institute of Health), Berlin, Germany; ^5^ Berlin Institute of Health, Berlin, Germany

**Keywords:** neurooncology, interdisciplinary work, metastasis (cancer metastasis), glioblastoma, radiation oncology, brain tumors

Neuro-oncology is a multidisciplinary field in rapid expansion in the recent years (1). Molecular biology and basic science research are providing many innovative insights toward the comprehension of the pathobiological mechanisms of nervous system tumors, from the malignancies, as glioblastomas, to the benign ones, as meningiomas, schwannomas and many others (1). On one hand, this is leading to the refinement of our pathological criteria to classify these tumors, as well as to significant advancements in the information that innovative diagnostic tools can provide (2). Indeed, neuroradiological and nuclear medicine techniques are currently characterized by an increasing predicting value of the morphological, structural, functional and metabolic features of the neoplasms and by the possibility to provide accurate and useful maps of brain eloquent cortical and subcortical areas in relationship to the tumor location, guiding its surgical resection to reduce the risk of possible complications or neurological sequelae (3, 4). On the other hand, the definition of the role of the different cellular interactions in the neoplasms and of their molecular pathways could represent the key of success to identify new therapeutical targets, potentially permitting medical and radiation oncologists to select the most specific treatment case by case, tailoring and personalizing their treatment on each single patient (1, 5).

The other side of the coin of this tremendous effort is represented by the growing difficulties for the different specialists involved in the treatment of these patients to share a common language. A harmful consequence of the large amount of data today available for every single patient, deriving for instance from the neuroradiological, pathological and molecular studies or from the surgical inspection, could be the degeneration of the multidisciplinary discussion about the most appropriate therapeutical strategy into a modern Tower of Babel, where the lack of an effective communication could lead to suboptimal choices.

An open and frank reflection about the methods that we use to manage our neuro-oncological patients can be useful to avoid this risk. Indeed, not only do basic, translational, or clinical research require an effective methodology to provide an appropriate answer to a specific investigation question, but also our routine clinical practice should be based on shared and multidisciplinary approved methods (1).

The aim of this Frontiers Research Topic was to include contributions from multiple specialists to analyze the diagnostic and therapeutic methods adopted both for benign or malignant nervous system tumors. The need to predict the survival of low-grade gliomas lead to the development of an exhaustive nomogram, with the aim to help the decision-making algorithm for these patients (Ao et al.). Indeed, although prognosis can be very variable in this cohort of cases, this study demonstrated that seven specific factors, as patients’ age, sex, histological type, extent of surgical resection, treatment with radiotherapy and with chemotherapy and tumor size have a significant role in determining the 3-, 5-, 10- years survival rates (Ao et al.). The impact of surgery on glioblastomas is examined by an innovative point of view, demonstrating how surgical complications can affect the subsequent therapies, remarking on the importance of the safety of every surgical maneuver available to our patients (Weber et al.). Indeed, Weber et al. demonstrated that peri-operative adverse events increases the time of initiation of adjuvant therapies and the risk of therapy interruption or non-initiation, with consequent detrimental effect on patients overall survival. Another study addressed gender dysmorphism in the diagnostics of gliomas using L-[S-methyl-11C-methionine (MET)-PET and its correlation with the prognostically relevant biomarker isocitrate dehydrogenase (IDH) mutation status, underlining the importance of gender-specific cohorts in establishing biomarkers for radiologic imaging (Papp et al.). First, there were no significant distinguishing features between female and male patients in either standardized uptake value or tumor-to-background ratio radiomics. However, female IDH+ patients had significant differences in the intensity histogram coefficient of variation (0.031) and mean intensity skewness (-0.327) compared with male IDH+ patients (0.068 and -0.123, respectively) (Papp et al.).

In benign tumors, a contrast-enhanced T1WI MRI sequence was evaluated to distinguish meningioma from schwannoma, exhibiting promising tumor-type-related diagnostic features as a new basis for preoperative differential diagnosis and treatment decisions (Cao et al.). Of note, the number of intratumoral microbleeds differed between meningiomas and schwannomas, with the number of microbleeds being significantly higher in schwannomas. Interestingly, there was also a distinction between epithelial meningiomas and fibrous meningiomas, with the number of microbleeds being higher in fibrous meningiomas but still much less than in schwannomas (Cao et al.). Finally, the interaction of a multidisciplinary team is presented in the management of a rare and interesting case of an adolescent with a supratentorial ependymoma, already treated with radiation and chemo-therapy after a gross tumor resection, who developed pulmonary metastasis after 5.3 years of disease-free survival without any local tumor recurrence, suggesting the effectiveness of adjuvant therapies on these replicative lesions (Xu et al.).

Overall, this section on methods in neuro-oncology contains five inspiring publications that encourage further studies on this Research Topic.

## Author contributions

MZ prepared the first draft. GA improved upon the initial draft. Both authors edited, reviewed, and approved the final version.

## References

[1] Barden MM Omuro AM. Top advances of the year: neuro-oncology. Cancer (2023) 129(10):1467–72. doi: 10.1002/cncr.34711 36825454

[2] WHO Classification of Tumours Editorial Board. World health organization classification of tumours of the central nervous system. 5th ed. Lyon: International Agency for Research on Cancer (2021).

[3] Mitolo M Zoli M Testa C Morandi L Rochat MJ Zaccagna F. Neuroplasticity mechanisms in frontal brain gliomas: a preliminary study. Front Neurol (2022) 13:867048. doi: 10.3389/fneur.2022.867048 35720068 PMC9204970

[4] Villani V Casini B Tanzilli A Lecce M Rasile F Telera S. The glioma-IRE project - molecular profiling in patients with glioma: steps toward an individualized diagnostic and therapeutic approach. J Transl Med (2023) 21(1):215. doi: 10.1186/s12967-023-04057-y 36959606 PMC10035236

[5] Ratti S Marvi MV Mongiorgi S Obeng EO Rusciano I Ramazzotti G. Impact of phospholipase c β1 in glioblastoma: a study on the main mechanisms of tumor aggressiveness. Cell Mol Life Sci (2022) 79(4):195. doi: 10.1007/s00018-022-04198-1 35303162 PMC8933313

